# PTBP1 Positively Regulates the Translation of Circadian Clock Gene, *Period1*

**DOI:** 10.3390/ijms21186921

**Published:** 2020-09-21

**Authors:** Wanil Kim, Jae-Cheon Shin, Kyung-Ha Lee, Kyong-Tai Kim

**Affiliations:** 1 Division of Cosmetic Science and Technology, Daegu Haany University, Hanuidae-ro 1, Gyeongsan, Gyeongbuk 38610, Korea; wkim@dhu.ac.kr; 2 Pohang Technopark, Pohang, Gyeongbuk 790-834, Korea; jcshin@ptp.or.kr; 3 Department of Life Sciences, Pohang University of Science and Technology, Cheongam-Ro 77, Pohang, Gyeongbuk 37673, Korea; 4 Division of Integrative Biosciences and Biotechnology, Pohang University of Science and Technology, Cheongam-Ro 77, Pohang, Gyeongbuk 37673, Korea

**Keywords:** PTBP1, Per1, circadian rhythm, IRES

## Abstract

Circadian oscillations of mRNAs and proteins are the main features of circadian clock genes. Among them, *Period1* (*Per1*) is a key component in negative-feedback regulation, which shows a robust diurnal oscillation and the importance of circadian rhythm and translational regulation of circadian clock genes has been recognized. In the present study, we investigated the 5′-untranslated region (5′-UTR) of the mouse core clock gene, *Per1*, at the posttranscriptional level, particularly its translational regulation. The 5′-UTR of *Per1* was found to promote its translation via an internal ribosomal entry site (IRES). We found that polypyrimidine tract-binding protein 1 (PTBP1) binds to the 5′-UTR of *Per1* and positively regulates the IRES-mediated translation of *Per1* without affecting the levels of *Per1* mRNA. The reduction of PTBP1 level also decreased the endogenous levels of the PER1 protein but not of its mRNA. As for the oscillation of PER1 expression, the disruption of PTBP1 levels lowered the PER1 expression but not the phase of the oscillation. PTBP1 also changed the amplitudes of the mRNAs of other circadian clock genes, such as *Cryptochrome 1* (*Cry1*) and *Per3*. Our results suggest that the PTBP1 is important for rhythmic translation of *Per1* and it fine-tunes the overall circadian system.

## 1. Introduction

Almost all living things on Earth are influenced by the day and night cycle and have a roughly 24-h rhythmic cycle of physiological processes. This physiological, 24-h cycle that displays an endogenous and entrainable oscillation is defined as “circadian rhythm” [1]. Circadian rhythm is important for efficient operation of biological functioning and for ensuring the quality of life. An obvious manifestation of circadian rhythmicity is in the sleeping and feeding patterns of animals, including humans. Distinct patterns are also observed for body temperature, blood pressure, brain waves, hormone production, cell regeneration and several other biological activities [2,3,4]. Disruption of circadian rhythm is closely related to cancer, abnormal hormone secretion, obesity, myocardial infarction and insomnia [5,6,7]. The diseases related to circadian rhythm can originate from any abnormal clock-controlled gene that is affected by dysregulation of the circadian rhythm. Many genes expressed in the suprachiasmatic nucleus of the hypothalamus and in some organs, such as the liver and heart, show circadian rhythm-dependent expression patterns [8,9,10]. It is estimated that the transcription of about 43% of all protein-coding genes in mice is under the control of circadian rhythm [11]. Most of these rhythmically accumulated mRNA pools result in rhythmic protein expression. However, some rhythmic protein patterns can originate from non-rhythmically transcribed mRNAs by rhythmic translation [12]. In fact, rhythmic posttranscriptional regulation and time-dependent posttranslational modifications have also been also reported [13,14,15]. Because circadian rhythm-dependent posttranscriptional regulation has been revealed to be common in many genes in mammalian genomes, it can be assumed that all genes are probably regulated by the circadian rhythm.

The circadian rhythm is the sum of individual rhythms of brain and peripheral cells, having the molecular clock system that is composed of feedback loops [16]. The BMAL1/CLOCK heterodimer induces the transcription of *Period*, *Cryptochrome*, *Rev-erb α* and *Ror α* [17]. The PERIOD and CRYPTOCHROME heterodimer represses the activation of BMAL1/CLOCK [18]. The mRNA and protein levels of these clock genes show a time-dependent expression. Maintenance of a rhythmic mRNA and protein profile involves time-dependent processes of transcription, mRNA degradation, initiation of translation and degradation of proteins. Among these clock genes, dramatic oscillation is observed for *Period1* (*Per1*), which is one of the well-known and important clock genes in the mammalian circadian system. The robust rhythmic amplitude of PER1 is generated by circadian time-dependent translation of *Per1* mRNA and involves an internal ribosomal entry site (IRES) in the 5′-untranslated region (5′-UTR) of the *Per1* mRNA [15]. Although the role of IRES was first reported for the expression of viral genes, many studies have shown that mammalian cells also utilize an IRES-mediated translation mechanism for rapid adaptation to certain environments, such as apoptosis [19], chemotoxic stress [20] and mitosis [21]. For IRES-mediated translation, proteins known as IRES trans-acting factors (ITAF) must interact with IRES elements in a structure- or sequence-dependent manner [22]. Previously, we reported that rhythmic cap-independent translation mediated by synaptotagmin-binding cytoplasmic RNA-interacting protein (SYNCRIP) is occurred on the IRES in mouse *Per1* 5′-UTR [15]. And the reduction of SYNCRIP expression decreased the amplitude of PER1 protein oscillation without change of the *Per1* mRNA oscillation pattern. The rhythmic binding of SYNCRIP to the *Per1* 5′-UTR region was critical for maintaining the circadian rhythmicity of PER1 protein.

Polypyrimidine tract-binding protein 1 (PTBP1), also known as heterogeneous nuclear ribonucleoprotein I (HNRNPI), is a nucleocytoplasmic shutting cellular RNA-binding protein that is abundant in many tissues [23,24]. PTBP1 binds to polypyrimidine-rich sequences of single-stranded RNAs [25] and has been implicated in alternative splicing [25,26] and modulation of polyadenylation efficiency of pre-mRNAs [27] as well as stability of mRNAs [28,29]. PTBP1 is a key ITAF modulating the activities of a large number of viral and cellular IRESs [26,30,31]. PTBP1 has been shown to remodels the secondary structure of the IRES and allowing the IRES to attain a correct structural conformation for ribosome binding [32].

In the present study, we confirmed our hypothesis that polypyrimidine tract-binding protein 1 (PTBP1) acts as an ITAF for the IRES-mediated translation of *Per1* and is necessary for the maintenance of the robust circadian amplitude of PER1 protein by enhancing its translation. Our study should expand the understanding of the roles of the 5′-UTR of clock mRNAs and RNA-binding proteins that interact with them in maintaining the biological clock through circadian regulation of translation.

## 2. Results

### 2.1. PTBP1 Activates the IRES Activity of Per1

**Figure 1:**
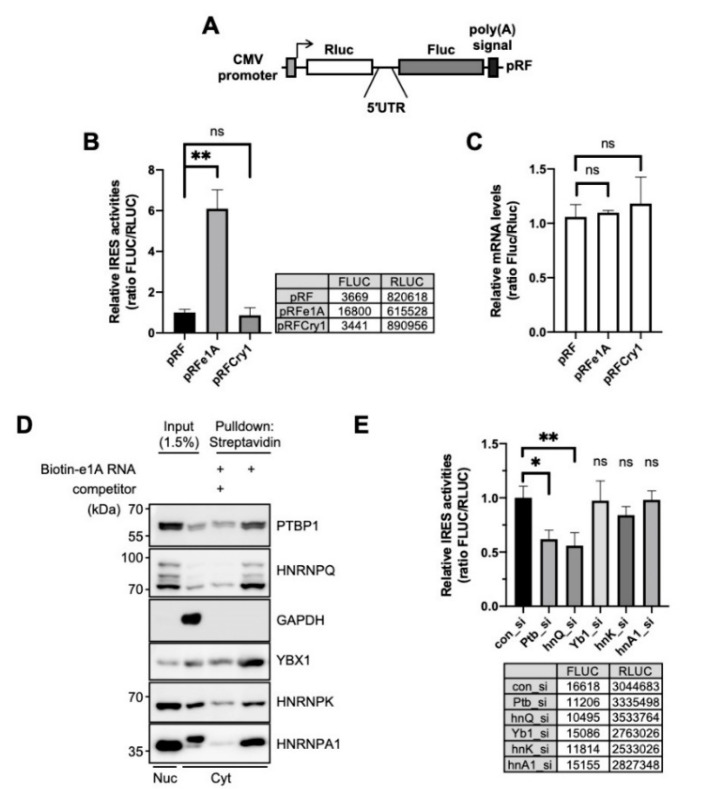
Polypyrimidine tract-binding protein 1 (PTBP1) is an ITAF of the *Per1* 5′-untranslated region (UTR). (**A**) Schematic diagram of bicistronic reporter plasmids containing the full-length *Per1* 5′-UTRs. The pRF bicistronic reporter plasmid (pRF). *Renilla* luciferase and firefly luciferase are shown. (**B**) HEK 293T cells were transiently transfected with bicistronic reporter plasmids and then luciferase activities were measured. The ratio of the activities obtained for the 5′-UTR-less control vector, pRF, was set to 1. *n* = 3 biological repeats; ** *p* < 0.01, as determined by one-way ANOVA with Tukey’s multiple comparison test. (**C**) Relative reporter mRNA levels were measured after the transfection of bicistronic reporter plasmids. The values obtained for pRF were set to 1. *n* = 3; statistical analysis was performed by one-way ANOVA with Tukey’s multiple comparison test. (**D**) The in vitro transcribed *Per1* 5′-UTR constructs were labeled with biotin-UTP and incubated with nuclear or cytoplasmic extracts of NIH 3T3 cells. After streptavidin-affinity purification, samples were separated by SDS-PAGE and subjected to immunoblotting with the indicated antibodies. Abundant RNA-binding proteins, PTBP1, HNRNPQ, YBX1, HNRNPK, HNRNPA1, were detected in the reaction with biotin-labeled mRNA but their binding decreased in the presence of 5-fold excess of non-labeled *Per1* 5′-UTR mRNA. (**E**) pRFe1A plasmids and indicated specific siRNAs were transfected into NIH 3T3 cells and then relative IRES activities were measured. Control siRNA, con_si; Ptbp1-specific siRNA, Ptb_si; hnRNP Q specific siRNA, hnQ_si; Y-box specific siRNA, Yb1_si; hnRNP K specific siRNA, hnK_si; hnRNP A1 specific siRNA, hnA1_si. *n* = 3; * *p* < 0.05, ** *p* < 0.01, as determined by one-way ANOVA with Tukey’s multiple comparison test.

We assessed and confirmed the IRES activity of *Per1* by using a bicistronic reporter system, which produces bicistronic *Renilla* luciferase (*Rluc*) and firefly luciferase (*Fluc*). The *Rluc* mRNA is translated in a cap-dependent manner but the *Fluc* region of the bicistronic mRNA is translated under the control of an intergenic 5′-UTR sequence between *Rluc* and *Fluc* (Figure 1A). Thus, the translation of *Fluc* reflects the IRES activity of the inserted intergenic sequences. *Per1* has two forms of 5′-UTRs (e1A and e1B) (Supplementary Figure S1A); however, both have identical sequences in the coding region of the transcript. These 5′-UTRs of the *Per1* mRNA are generated by alternative promoter usage [18]. The e1A 5′-UTR of *Per1* showed significantly higher (~6–7-fold) IRES activity than the control (pRF) or the 5′-UTR of *Cry1* (Figure 1B). The IRES activity of e1B was also significant and was similar to that of e1A (Supplementary Figure S1B) and the potential location of the IRES might be the region between 144 and 63 of *Per1* 5′-UTR [15] (Supplementary Figure S1A). We also assessed the mRNA levels of the reporters to exclude the possibility that the higher IRES activity was due to the increased mRNA levels. The levels of reporter mRNAs were similar for the control and 5′-UTR-inserted constructs (Figure 1C). From these results, we conclude that the 5′-UTR of *Per1* has IRES activity that is involved in increasing the protein level and this phenomenon might originate from the upregulation of IRES-mediated translation and not as a result of any change in the mRNA levels.

IRES-mediated translation, which is a noncanonical type of translation of genes, is typically regulated by ITAFs. An ITAF binds to mRNA and controls the translation system by stabilizing the conformation of RNA structure or recruiting factors of the ribosome [33,34]. To identify ITAF candidates that might regulate the translation of *Per1*, many RNA-binding proteins were analyzed for their interaction with the 5′-UTR of *Per1* using biotin-RNA affinity precipitation. We found several *Per1* 5′-UTR-interacting RNA-binding proteins, namely polypyrimidine tract-binding protein 1 (PTBP1), SYNCRIP, Y-box protein 1 (YBX1), heterogeneous nuclear ribonucleoprotein K (HNRNPK) and heterogeneous nuclear ribonucleoprotein A1 (HNRNPA1). The identified RNA-binding proteins co-precipitated with biotin-labeled *Per1* 5′-UTR RNA; the binding affinities were, however, markedly reduced upon addition of non-labeled *Per1* 5′-UTR competitors (Figure 1D). To understand the function and effect of the identified RNA-binding proteins on the *Per1* IRES activity, the IRES activity was assessed after the knockdown of these proteins. Among the selected RNA-binding proteins, the knockdown of *Ptbp1* achieved by specific small interfering RNA (siRNA) (Ptb_si) attenuated the IRES-mediated translation of *Per1* to about 40% (Figure 1E). The knockdown of *Syncrip* (hnQ_si) also reduced the IRES-mediated translation of *Per1*, as previously reported [15] but those of *Y-box protein 1* (Yb1_si), *HnrnpK* (hnK_si) and *HnrnpA1* (hnA1_si) did not result in significant changes [15]. Based on these results, we conclude that PTBP1 binds to the *Per1* 5′-UTR and significantly contributes to the translation of *Per1* mRNA.

### 2.2. PTBP1 Controls the IRES Activity of Per1 and Consequently the PER1 Levels

**Figure 2:**
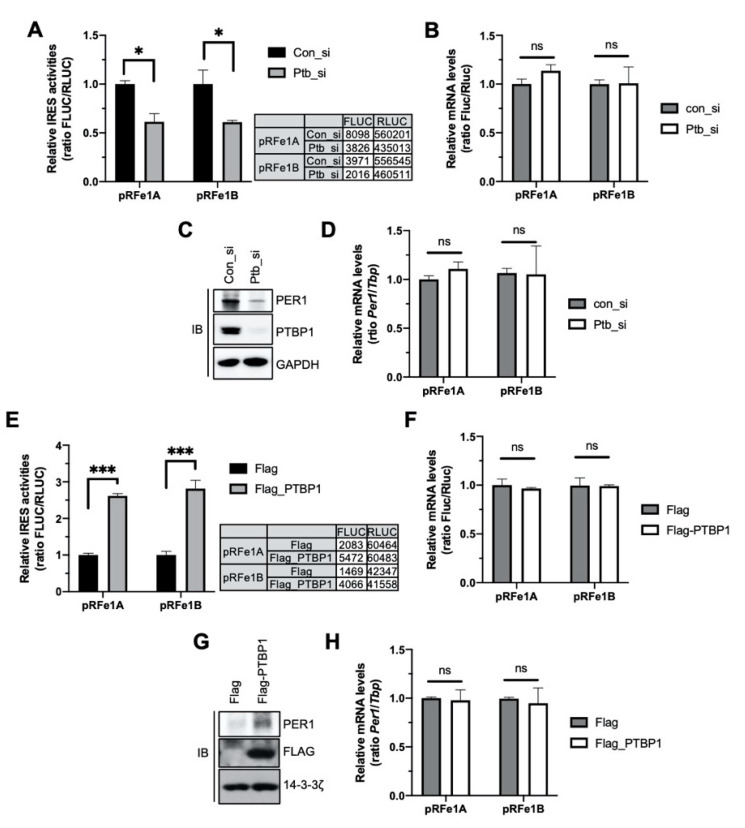
Polypyrimidine tract-binding protein 1 (PTBP1) controls the internal ribosomal entry site (IRES) activity of *Per1* and the PER1 protein levels. (**A**) Bicistronic plasmids, pRFe1A and pRFe1B, containing the *Per1* 5′-untranslated regions (UTRs) were transfected into cells with control or Ptbp1-specific siRNAs. These cells were used for measuring the luciferase activities; the relative IRES activities are shown. *n* = 3; * *p* < 0.05, as determined by two-way ANOVA with Sidak’s multiple comparison test. (**B**) From the cells mentioned for panel A, total RNA was prepared and real-time polymerase chain reaction (PCR) was performed with *Fluc-* and *Rluc*-specific primers. *n* = 3; two-way ANOVA with Sidak’s multiple comparison test was performed. Two variables are ‘type of *Per1* 5′UTRs’ and ‘con_si or Ptb_si’. (**C**) The knockdown of *Ptbp1* shown in panel A was confirmed by immunoblotting with the indicated antibodies. Endogenous PER1 protein levels were also assessed. The blots were cropped from the different parts of the same gel. (**D**) Relative endogenous *Per1* mRNA levels in the samples used for panel B were measured by real-time PCR. *n* = 3; two-way ANOVA with Sidak’s multiple comparison test was performed. (**E**) Bicistronic plasmids harboring the *Per1* 5′-UTRs were transfected with Flag or Flag-PTBP1 into HEK 293 cells. After 24 h of incubation, the cells were subjected to luciferase assay. *n* = 3; *** *p* < 0.001, as determined by two-way ANOVA with Sidak’s multiple comparison test. (**F**) Total RNA was prepared from the cells mentioned for panel E and used for real-time PCR with *Fluc-* and *Rluc*- specific primers. *n* = 3; two-way ANOVA with Sidak’s multiple comparison test was performed. (**G**) Overexpression of PTBP1 shown in panel E and endogenous PER1 protein levels were checked by immunoblotting by using the same extract as mentioned for panel E. Endogenous *Per1* mRNA levels were quantitated using the same samples as mentioned for panel F. The blots were cropped from the different parts of the same gel and incubated with different antibodies and for different times. (**H**) Relative endogenous *Per1* mRNA levels were measured after PTBP1 overexpression. *n* = 3; two-way ANOVA with Sidak’s multiple comparison test was performed.

To identify the function of PTBP1 in *Per1* IRES-mediated translation, the IRES activity of the *Per1* 5′-UTR was analyzed after knockdown of *Ptbp1*. Both the *Per1* 5′-UTRs showed reduced IRES activities upon knockdown of *Ptbp1* (Figure 2A). The mRNA levels of the *Per1* 5′-UTR reporter were also assessed to determine whether the reduction in the IRES activities of the *Per1* 5′-UTRs after the knockdown of *Ptbp1* was mediated by decreased IRES-mediated translation or by the altered mRNA levels of the *Per1* 5′-UTR reporter. As shown in Figure 2B, the knockdown of *Ptbp1* did not change the mRNA levels of the reporter (Figure 2B). The PER1 protein level, as well as the *Per1* IRES activity, decreased after the *Ptbp1* knockdown (Figure 2C and Supplementary Figure S2A). As correlated with the reporter mRNAs, the *Ptbp1* knockdown did not alter the endogenous levels of *Per1* mRNA, although it reduced the levels of the PER1 protein (Figure 2D). The knockdown of *Hnrnp A1* or *Hnrnp K*, both of which are also *Per1* 5′-UTR mRNA binding proteins but did not affect the IRES activity of *Per1* (Figure 1E), did not significantly reduce the levels of the PER1 protein (Supplementary Figure S2B).

In contrast, the overexpression of Flag-tagged PTBP1 increased the IRES activities of the *Per1* 5′-UTRs and consequently the levels of the PER1 protein (Figure 2E,G). The overexpression of PTBP1 did not change the levels of the *Per1* 5′-UTR reporter mRNA and *Per1* mRNA (Figure 2F,H). These data indicate that PTBP1 only controls the translation of *Per1* mRNA without causing any change in the mRNA levels.

### 2.3. PTBP1 Specifically Modulates the IRES Activity of Per1

**Figure 3:**
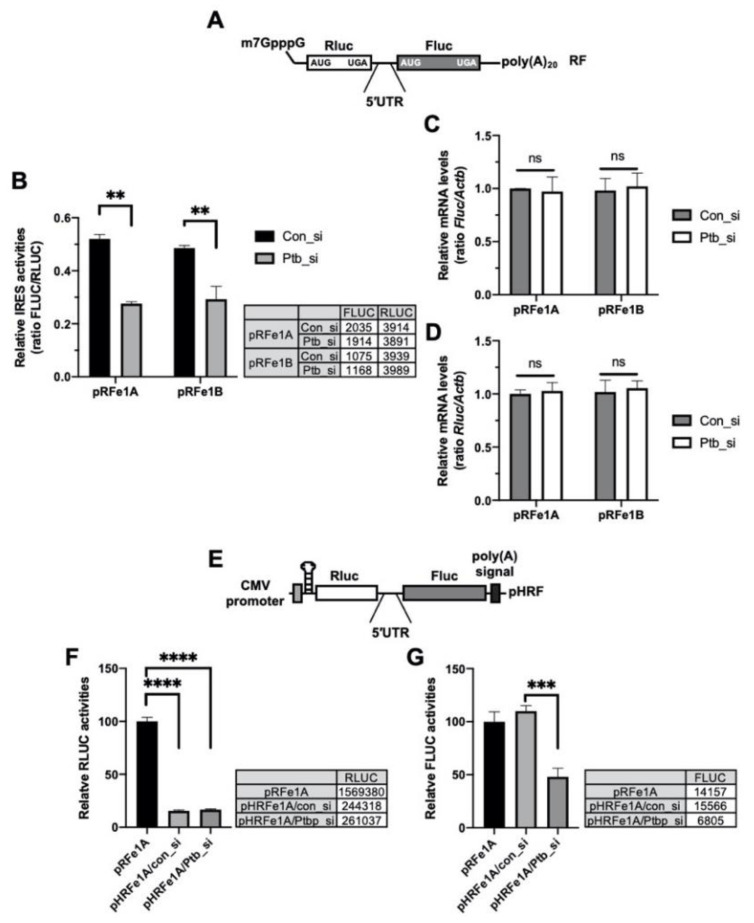
Polypyrimidine tract-binding protein 1 (PTBP1) functions as an IRES-transacting factor (ITAF) of the *Per1* mRNA. (**A**) Schematic diagram of the bicistronic mRNA reporter containing the *Per1* 5′-untranslated regions (UTRs). (**B**) con_si or Ptb_si was transfected into NIH 3T3 cells and incubated for 12 h. Subsequently, the cells were transfected with the *Per1* mRNA reporters. After incubation for 6 h, the cells were used for the luciferase assay. *n* = 3; ** *p* < 0.01, as determined by two-way ANOVA with Sidak’s multiple comparison test. (**C**,**D**) Relative mRNA levels in the same sample mentioned for panel B were checked by real-time PCR with the indicated specific primers. *n* = 3; two-way ANOVA with Sidak’s multiple comparison test was performed. (**E**) The bicistronic vector harboring a hairpin and loop with the *Per1* 5′-UTRs. (**F**,**G**) The hairpin-inserted reporter constructs were transfected into NIH 3T3 cells and these cells were used for luciferase assay. The luciferase activities of the pRF vector containing the *Per1* 5′-UTR e1A (RLUC, panel F; FLUC, panel G) was set to 100. *n* = 3; **** *p* < 0.0001, *** *p* < 0.001, as assessed by one-way ANOVA with Tukey’s multiple comparison test.

The 5′-UTR DNA sequence can contain a cryptic promoter or splicing acceptor site that creates a monocistronic transcript of the downstream region [35]. To rule out these transcriptional or posttranscriptional effects of the 5′-UTRs, bicistronic reporter mRNAs containing the 5′-m7GpppG cap structure for e1A and e1B [15] (Figure 3A) were transfected into cells, with or without an siRNA against *Ptbp1* and the IRES activities were assessed. The knockdown of *Ptbp1* decreased the IRES activities of the reporter mRNA system (Figure 3B); however, the reporter mRNA levels were not altered and the data were similar to those shown in Figure 2B (Figure 3C,D). The translation of the second cistron in the bicistronic system could be accelerated as a result of the read-through of ribosome through the intergenic region containing the 5′-UTR. To determine whether the effect of PTBP1 on the *Per1* 5′-UTR, with regard to the facilitation of translation of the second cistron, was due to ribosome reinitiation, a synthetic hairpin loop was inserted upstream of the *Rluc* in the pHRF reporter vector (Figure 3E). The insertion of the hairpin loop structure reduced the expression of the *Rluc*, upstream of the 5′-UTR as the first cistron, by over 80% compared to the hairpin loop-free pRF reporter (Figure 3F). The knockdown of *Ptbp1* did not change the *Rluc* expression. The insertion of the hairpin loop also did not affect the expression of FLUC relative to the pRF control vector (Figure 3G). However, the knockdown of *Ptbp1* decreased the expression of the *Fluc* but not of the *Rluc*. This means that the decrease in the IRES activities upon PTBP1 knockdown (Figure 2A) is not the result of decreased ribosome reinitiation in the bicistronic reporter system. From these results, we conclude that modulation of the *Per1* IRES activities by PTBP1 is not influenced by the cryptic promoter activity of the *Per1* 5′-UTR (Figure 3B) or ribosome reinitiation by PTBP1 (Figure 3E).

### 2.4. PTBP1 Does Not Affect the Expression of Per1 mRNA

**Figure 4:**
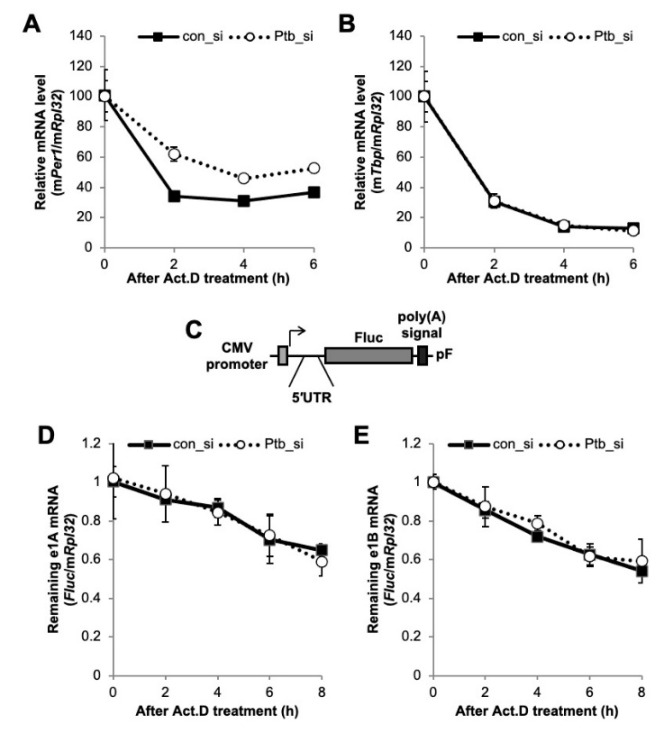
The function of polypyrimidine tract-binding protein 1 (PTBP1) on the *Per1* mRNA. (A and B) Con_si or Ptb_si transfected NIH 3T3 cells were treated with actinomycin D (Act. D) and harvested at the indicated time points. Relative endogenous mRNA levels of (**A**) *Per1* and (**B**) *Tbp* were determined by real-time PCR and normalized to the *Rpl32* mRNA levels at the indicated time points. (**C**) Schematic diagram of the *Per1* 5′-UTR reporter system. (**D**,**E**) The *Per1* 5′-UTR reporters were transfected into NIH 3T3 cells with con_si or Ptb_si. After 24 h of incubation, the cells were treated with Act. D and harvested at the indicated time points. Relative *Fluc* mRNA levels were determined with respect to the endogenous *Rpl32*. The initial relative mRNA levels of con_si were set to 100. *n* = 4; the values are means ± standard error of mean (SEM).

We showed that PTBP1 modulates the IRES-mediated translation of the *Per1* 5′-UTR. The RNA-binding protein, PTBP1, is well known for its major function in splicing and mRNA degradation [33,36,37]. Indeed, the 5′-UTR is also an important regulatory region for mRNA degradation and translation control [14,38]. Therefore, we checked the possibility that PTBP1-mediated regulation of IRES activities of the *Per1* 5′-UTR could be due to changes in the stability of the *Per1* mRNA at different PTBP1 expression levels. To check the mRNA stability, except for transcriptional supplementation, the *Per1* mRNA level was determined after actinomycin D (Act. D) treatment, which blocks the elongation step during transcription [39], so that remnant mRNA level without any new synthesis could be measured. The knockdown of *Ptbp1* slightly increased the *Per1* mRNA degradation kinetics compared to the degradation observed upon transfection with the control siRNA (Figure 4A). However, the stability of the mRNA of another control gene, *Tbp*, did not change in accordance with the *Ptbp1* expression levels (Figure 4B). The knockdown of *Ptbp1* did not increase the *Per1* mRNA levels as shown in Figure 2D, although the stability of the *Per1* mRNA was slightly increased (Figure 4A). To rule out any posttranscriptional regulation via the coding region and the 3′-UTR of *Per1*, we checked the mRNA stability with the reporter construct containing only the *Per1* 5′-UTR (Figure 4C). After the transfection of the 5′-UTR reporter constructs into cells, the stability of the reporter mRNA was determined upon *Ptbp1* expression. The mRNA stability of both the *Per1* 5′-UTRs, e1A and e1B, was not affected by *Ptbp1* expression (Figure 4D,E). These results indicate that *Ptbp1* modulates the IRES-mediated translation of *Per1* mRNA without causing changes in the stability of the *Per1* 5′-UTR mRNA as well as in the levels of the *Per1* mRNA. This implies a specific role for PTBP1 as an ITAF for the *Per1* mRNA.

### 2.5. PTBP1 Affects the Circadian Expression of Per1

**Figure 5:**
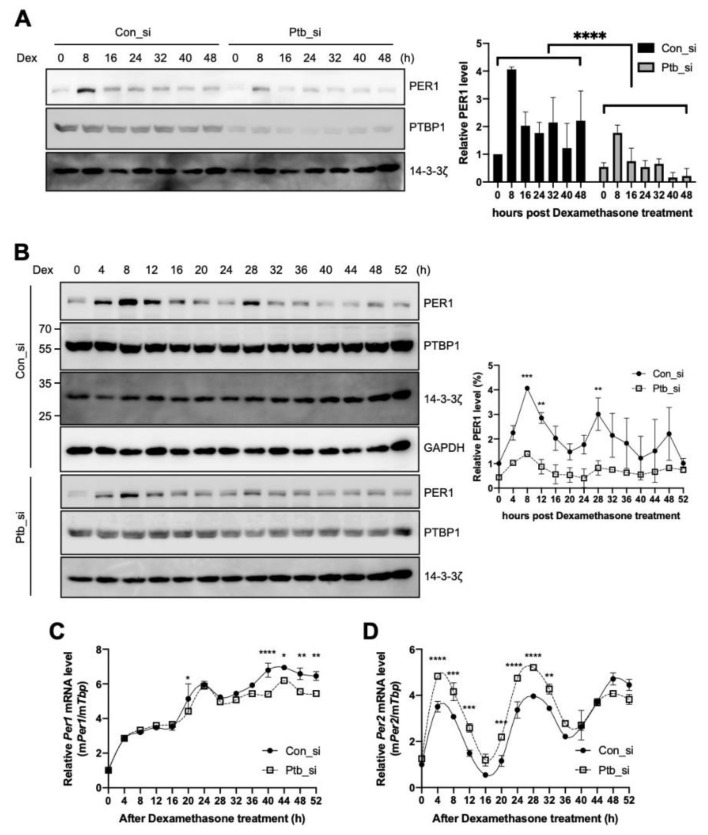
Polypyrimidine tract-binding protein 1 (PTBP1) regulates the circadian rhythm of the cell. (**A**,**B**) NIH 3T3 cells were transfected with siRNAs targeting *Ptbp1* or with control siRNA by microporation, incubated for 12 h, treated with dexamethasone and harvested at the indicated time points. Thereafter, immunoblotting was performed with the indicated antibodies. Blots of con_si and Ptb_si were prepared from different gels and all the con_si or Ptb_si annotated blots were cropped from the different parts of the same gel. These were then incubated with different antibodies for different times. The relative levels of PER1 were calculated and plotted. *n* = 2; **** *p* < 0.0001, *** *p* < 0.001, ** *p* < 0.01, as determined by two-way ANOVA with Sidak’s multiple comparison test. (**C**,**D**) Total RNA was prepared from the harvested cells for which data are shown in panel B and then the relative levels of indicated mRNAs were determined by real-time PCR. *n* = 3; **** *p* < 0.0001, *** *p* < 0.001, ** *p* < 0.01, * *p* < 0.05 as determined by two-way ANOVA with Sidak’s multiple comparison test.

We observed that PTBP1 modulates the IRES activities of *Per1* and consequently the protein levels of PER1 (Figure 2A,C). To determine the function of PTBP1 on *Per1* in terms of circadian physiology, *Ptbp1* was knocked down and the expression levels of *Per1* were checked after circadian synchronization done by dexamethasone treatment. As shown in Figure 2C, the knockdown of *Ptbp1* decreased the PER1 protein levels at all the time points (Figure 5A). The knockdown of *Ptbp1* decreased the expression of PER1; however, it did not alter the oscillation phase of PER1 (Figure 5B). The expression of PER1 as shown in Figure 5C was analyzed by the cosinor model and the presence and significance of oscillations was evaluated [40] (Supplementary Figure S3A–C). The reduced PTBP1 levels did not decrease the levels of endogenous *Per1* mRNA (Figure 2D) and the phenotype was also manifested in synchronized circadian rhythm analysis (Figure 5C). To understand the overall effects of *Ptbp1* in the circadian clock system, expression patterns of other clock genes were also examined because changes in PER1 protein can regulate the feedback loop of the core clock. The knockdown of *Ptbp1* increased the mRNA levels of *Per2* (Figure 5D) and *Arntl* (Supplementary Figure S4A). The reduced expression of *Ptbp1* decreased the mRNA levels of *Per3*, *Nr1d1* and *Cry1* after 24 h of dexamethasone treatment (Supplementary Figure S4B–D). However, *Ptbp1* did not alter the expression level or phase of *Dbp* mRNA (Supplementary Figure S4E). Taken together, *Ptbp1* regulates the translation of *Per1* via noncanonical IRES-mediated translation and it may be responsible for changes in the other circadian genes as well.

## 3. Discussion

In this study, we found that PTBP1 interacted with the 5′-UTR of *Per1* and increased the IRES-mediated translation of the *Per1* without causing changes in the stability of the *Per1* mRNA. Although knockdown of PTBP1 did not affect the mRNA stability of *Per1* in our luciferase reporter system after Act. D treatment (Figure 4D), decreased expression of PTBP1 slightly increased the mRNA stability of the endogenous whole *Per1* transcript (Figure 4A). We also found that the knockdown or overexpression of *Ptbp1* did not change the mRNA levels of the *Per1* mRNA, which implies that the overall *Per1* mRNA level was also regulated by other mechanisms (Figure 2D,H). Increased stability of the *Per1* mRNA under the *Ptbp1* knockdown condition might have been due to the function of other parts, presumably the 3′-UTR of *Per1* rather than the 5′-UTR. The 3′-UTR regulatory regions can influence the translation efficiency, localization, polyadenylation and stability of mRNAs [41]. The 3′-UTR of *Per1* is also an important regulatory region for its posttranscriptional regulation [42]. The relationship between the levels of *Per1* mRNA and protein could be clearly explained with further in-depth investigations into RNA-binding proteins, untranslated regions and their complex relationship.

The knockdown of *Ptbp1* decreased the rhythmic expression of the PER1 protein and changed the mRNA expression pattern of *Per2*, *Arntl*, *Nr1d1*, *Per3* and *Cry1*, although PTBP1 knockdown did not affect the mRNA levels of the *Per1* 5′-UTR reporter or endogenous *Per1* (Figure 2B,D and Figure 4D). We only assessed the binding of PTBP1 with the *Per1* mRNA and also its function. PTBP1 is a ubiquitously expressed heterogeneous nuclear ribonucleoprotein (hnRNP) that forms complexes with other hnRNPs and mRNAs. Indeed, the molecular circadian clock system is interlocked. Therefore, it is difficult to say that the changed expression patterns of several clock gene mRNAs, when *Ptbp1* was knocked down, are directly due to the altered expression of PER1 or PTBP1 protein. However, our study partially clarifies the function of PTBP1 in the noncanonical translation of the *Per1* mRNA.

RNA-binding proteins have several functions. SYNCRIP, which functions as an ITAF, was shown to rhythmically accelerate mRNA degradation and increase IRES-mediated translation of several target genes [15,43]. PTBP1 is also known as one of the components of pre-mRNA splicing machinery, which is a multi-component complex necessary for the splicing step [44]. PTBP1 also activates mRNA degradation but increases the translation of specific target genes [29,45]. These opposite and varied functions of RNA-binding proteins are based on the context of the interacting proteins. RNA-binding proteins generally bind to the target RNAs and recruit their interacting proteins in different ways based on the cellular context. Context-dependent interaction of RNA-binding proteins with target transcripts leads to diverse consequences, resulting in functional divergence. For the regulation of *Per1* IRES-mediated translation, many factors might be involved, such as other ITAFs and ribosomal proteins. SYNCRIP, a previously reported ITAF of *Per1* and PTBP1 might recruit several different types of translation initiation machinery to fully induce IRES-mediated translation. Otherwise, each ITAFs could be a different switch for the translation initiation. Binding of RNA to SYNCRIP is specifically inhibited by its phosphorylation and it might regulate the mRNA translation or stability [46]. Methylation of SYNCRIP is also important for its nuclear localization [47]. Indeed, the amounts of SYNCRIP bound to IRES can be increased under heat stress conditions [48]. In the case of PTBP1, transport of PTBP1 can be modulated by its phosphorylation [24] and PTBP1 has a role in determining mRNA localization in the cytoplasm [49]. PTBP1 also has been suggested that it may act as an RNA chaperone to help IRES attain optimal conformation for the translation initiation or the binding of other ITAFs [32,33]. Moreover, it has been reported that several protein kinases are under the control of circadian rhythm or can regulate circadian rhythm [50,51]. To elucidate the roles of a given RNA-binding protein, context-dependent approaches should be considered. And the relationship between the post-translational modification of RNA binding proteins and IRES activity might clarify the complex function of ITAFs for each gene.

The present study is the first to reveal the functional role of PTBP1 in the 5′-UTR-mediated *Per1* translation in the circadian system. The present study also elucidates a new mechanism of rhythmic 5′-UTR-mediated *Per1* translation, which may be an important step in the regulation of the circadian clock. From these results, we conclude that posttranscriptional regulation by RNA-binding protein, PTBP1, plays an important role in the rhythmic expression of *Per1*. The present study might help explain the regulation of the amplitude of clock genes in the circadian rhythm. We hope that our findings may reveal hidden crucial aspects of the complex molecular system for achieving the tightly regulated 24-h cycle in mammals, with a distinct translation activation mechanism.

## 4. Materials and Methods 

### 4.1. Plasmid Constructs

The pRF, hairpin loop inserted pHRF and pSK′ constructs used for in vitro binding assay and the pCY2 system for mRNA reporters harboring the mouse *Per1* 5′-UTRs (e1A and e1B) were used as previously reported [15,22]. 

### 4.2. Cell Culture and Drug Treatment

NIH 3T3 (Korean Cell Line Bank, Seoul, Korea) or HEK 293T (ATCC, Manassas, VA, USA) cells were cultured in Dulbecco’s modified Eagle’s medium (HyClone) supplemented with 10% fetal bovine serum (HyClone) and 1% antibiotics (HyClone) and maintained in a humidified incubator with 95% ambient air and 5% of CO_2_ at 37 °C.

The circadian oscillation of NIH 3T3 cells was synchronized by treatment with 100 nM dexamethasone (Sigma-Aldrich). After 2 h, the medium was replaced with complete medium [15,52,53]. For blocking the transcription, NIH 3T3 cells were treated with 5 μg/mL of actinomycin D (Sigma-Aldrich) [14,54]. 

### 4.3. Transient Transfection and RNA Interference

For transient transfection of the reporter plasmids, specific small interfering RNAs (siRNAs) and PTBP1-overexpressing plasmids in NIH 3T3 cells, the Neon^®^ Transfection System (Invitrogen) was used as recommended by the manufacturer.

The reporter mRNA transfection was performed as follows—NIH 3T3 cells were transiently transfected with 2 μg of the in vitro transcribed reporter mRNAs containing the cap structure at the 5′-end using Lipofectamine 2000 (Invitrogen) followed by incubation for 6 h before harvesting [14].

### 4.4. In Vitro RNA Synthesis, In Vitro Binding

For in vitro binding assays, biotin-labelled RNA was transcribed from *Xba*I-linearized recombinant pSK′ vectors using T7 RNA polymerase (Promega). Streptavidin-biotin RNA affinity purification of the *Per1* 5′-UTR-binding proteins was performed as previously reported [14]. Briefly, cytoplasmic extracts prepared from NIH 3T3 cells were incubated with biotinylated *Per1* 5′-UTR and the resin-bound proteins were pulled down using streptavidin resin. Resin-bound proteins were analyzed by sodium dodecyl sulfate polyacrylamide gel electrophoresis (SDS-PAGE).

### 4.5. RNA Quantification

Total RNA was extracted from NIH 3T3 cells using the TRIzol (Invitrogen). RNA was reverse-transcribed using GoScript^TM^ Reverse Transcription Mix and oligo (dT) primer (Promega) according to the manufacturer’s instructions. The mRNA levels of endogenous genes or reporter plasmids were detected by quantitative real-time PCR using the StepOnePlus or QuantStudio 3 real-time PCR system (Applied Biosystems) with the FastStart Universal SYBR Green Master (Roche) or SYBR^TM^ Green PCR Master Mix (Applied Biosystems^TM^). Specific primer pairs for *Rluc*, *Fluc*, mouse *Actb*, mouse *Tbp*, mouse *Per1,* mouse *Cry1*, mouse *Rpl32*, mouse *Per2*, mouse *Nr1d1* and mouse *Dbp* were used for real-time PCR (the primer sequences are shown in Supplementary Table S1).

### 4.6. Immunoblot Analysis

Immunoblot analyses were performed using polyclonal anti-PER1, monoclonal anti-hnRNP Q (Sigma-Aldrich), monoclonal anti-PTBP1 (Abcam), monoclonal anti-YBX1 (Abcam), polyclonal anti-hnRNP K (Abcam), monoclonal anti-hnRNP A1 (Abcam), monoclonal anti-FLAG (Sigma-Aldrich), polyclonal anti-14-3-3ζ (Santa Cruz Biotechnology) and monoclonal anti-GAPDH (Millipore) as primary antibodies. Horseradish peroxidase-conjugated species-specific secondary antibodies (goat, Santa Cruz Biotechnology; guinea pig, Santa Cruz Biotechnology; mouse, Thermo Fisher Scientific; rabbit, Jackson ImmunoResearch Laboratories) were visualized by using a SUPEX ECL solution kit (Neuronex) or a Pierce^TM^ ECL Western Blotting substrate (Thermo Fisher Scientific) under LAS-4000 chemiluminescence detection system (FUJIFILM) and a FUSION Solo S (Vilber). The acquired images were analyzed according to the manufacturer’s instructions.

### 4.7. Statistical Analysis

Statistical parameters, including the definitions and exact values, are reported in the figures and the corresponding legends. Statistical analysis was performed using GraphPad Prism or Excel.

## Supplementary Materials

Supplementary materials can be found at https://www.mdpi.com/1422-0067/21/18/6921/s1.

## Abbreviations

Per1	Period1
UTR	untranslated region
IRES	internal ribosomal entry site
PTBP1	polypyrimidine tract-binding protein 1
Cry1	Cryptochrome 1
ITAF	IRES trans-acting factors
Rluc	Renilla luciferase
Fluc	firefly luciferase
Act. D	Actinomycin D
