# Supplementary material for: PTBP1 Positively Regulates the Translation of Circadian Clock Gene, Period1
Source: Int J Mol Sci. 2020 Sep 21;21(18):6921. doi: 10.3390/ijms21186921 (PMC7555454; doi:10.3390/ijms21186921)
Supplement: Supplementary file 1 [file ijms-21-06921-s001.pdf]

International Journal of *Molecular Sciences*

MDPI

Supplementary information

PTBP1 Positively Regulates the Translation of Circadian Clock Gene, *Period1*

Wanil Kim [1], Jae-Cheon Shin [2], Kyung-Ha Lee [1,*] and Kyong-Tai Kim [3,4,*]

[1] Division of Cosmetic Science and Technology, Daegu Haany University, Hanuidae-ro 1, Gyeongsan, Gyeongbuk 38610, Korea; wkim@dhu.ac.kr
[2] Pohang Technopark, Pohang, Gyeongbuk 790-834, Korea; jcshin@ptp.or.kr
[3] Department of Life Sciences, Pohang University of Science and Technology, Cheongam-Ro 77, Pohang, Gyeongbuk 37673, Korea
[4] Division of Integrative Biosciences and Biotechnology, Pohang University of Science and Technology, Cheongam-Ro 77, Pohang, Gyeongbuk 37673, Korea
* Correspondence: kyungha.lee@dhu.ac.kr (K.-H.L.); ktk@postech.ac.kr (K.-T.K.); Tel.: 82-53-819-7743 (K.-H.L.); 82-54-279-2297 (K.-T.K.)

Received: 24 August 2020; Accepted: 19 September 2020; Published: 21 September 2020

Keywords: PTBP1; Per1; circadian rhythm; IRES

1. Supplementary Figure

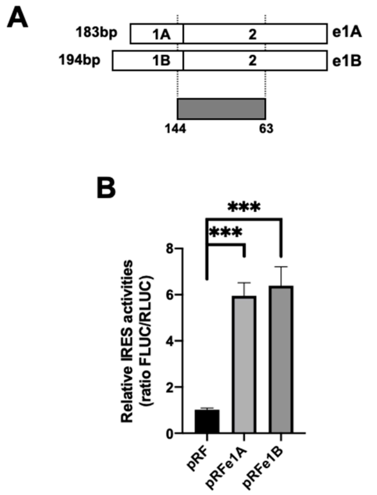

Supplementary Figure S1. (A) Schematic diagram of *Per1* 5′-UTRs and potential IRES region (gray). (B) IRES activity of *Per1* 5′-UTRs. Bicistronic plasmids, pRFe1A and pRFe1B, were transfected into cells. After incubation for 24 h, the cells were used for the luciferase assay. n = 3, *** $p < 0.001$, as determined by one-way ANOVA with Tukey's multiple comparison test.

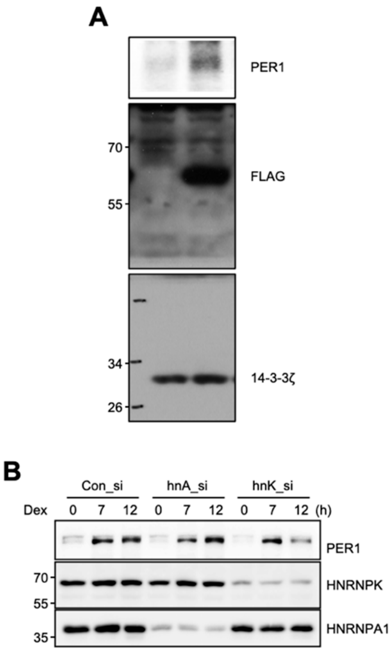

Supplementary Figure S2. (A) Complete immunoblots of the corresponding Figure, Fig. 2C. (B) Knockdown *Hnrnp A1* and *Hnrnp K* did not change PER1 protein levels. Control siRNA (Con_si) or gene-specific siRNAs for *Hnrnp A1* (hnA_si) or *Hnrnp K* (hnK_si) were transfected into NIH 3T3 cells. After a 12-h incubation, cells were treated with Dexamethasone (Dex), and were harvested at the indicated time points and subjected to immunoblotting with indicated antibodies.

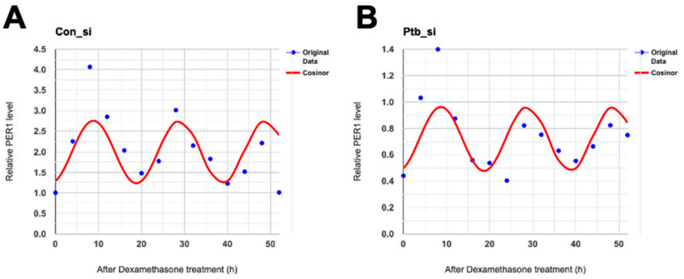

C

	Mesor	Amplitude	Acrophase (hours)	Bathyphase (hours)	P-value (zero-amplitude test)
**Con_si**	1.994	0.760	8.805	18.805	0.04187
**Ptb_si**	0.719	0.242	8.692	18.692	0.03084

Supplementary Figure S3. (A and B) The data of Figure 5B was analyzed by the cosinor model to evaluate the presence and significance of oscillations. (C) Table of cosinor analysis and zero-amplitude.

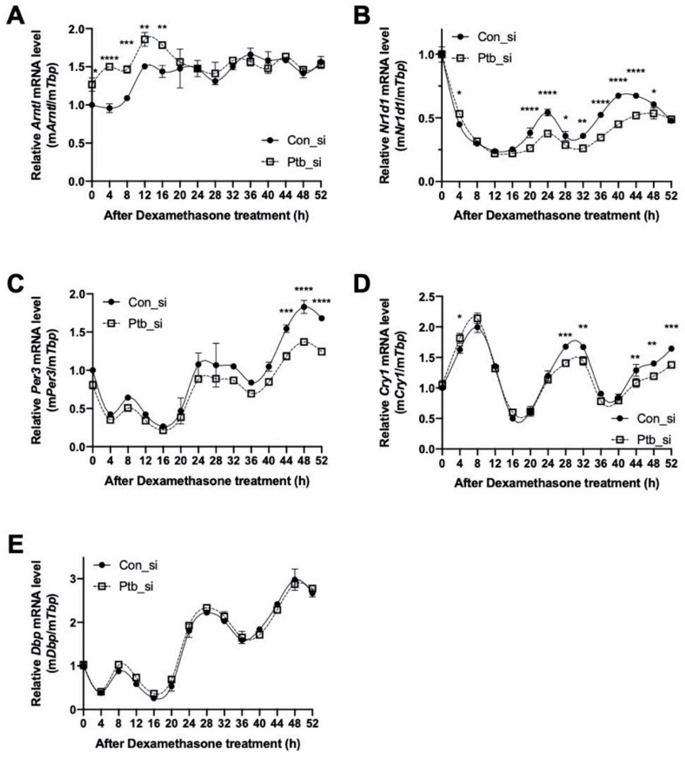

Supplementary Figure S4. PTBP1 modulates overall circadian rhythm. (A to E) The same extracts used for Figures 5B and 5C were subjected to real-time polymerase chain reaction. The relative mRNA levels of transfected control siRNA (closed circles/solid line) or *Ptbp1*-specific siRNA (open squares/dotted line) were shown. mRNA levels of (A) *Arntl*, (B) *Nr1d1*, (C) *Per3*, (D) *Cry1* and (E) *Dbp* were normalized to *Tbp* amounts, and the relative levels at 0 time were set to 1. Results shown are average values with the SEM. n = 3; * $p < 0.05$, ** $p < 0.01$, *** $p < 0.001$, **** $p < 0.0001$, as determined by two-way ANOVA with Sidak's multiple comparisons test.

2. Supplementary Table

Supplementary Table S1. Sequences of real-time PCR primers.

Gene	Forward or Reverse	Sequence (5' to 3')
Rluc	forward	GTAACGCTGCCTCCAGCTAC
	reverse	CCAAGCGGTGAGGTACTTGT
Fluc	forward	GAGGTTCCATCTGCCAGGTA
	reverse	CACACAGTTCGCCTCTTTGA
Actb	forward	TGTTACCAACTGGGACGACA
	reverse	GGGGTGTTGAAGGTCTCAAA
Tbp	forward	GGGGAGCTGTGATGTGAAGT
	reverse	CCAGGAAATAATTCTGGCTCAT
Per1	forward	CAGGCTAACCAGGAATATTACCAGC
	reverse	CACAGCCACAGAGAAGGTGTCCTGG
Cry1	forward	CCTTGAAAAGCCTGGGAAAT

	reverse	TCCGCTGCGTCTATATCCTC
Rpl32	forward	AACCCAGAGGCATTGACAAC
	reverse	CACCTCCAGCTCCTTGACAT
Per2	forward	AGGATGTGGCAGGTAACAGG
	reverse	ATGCTCCAAACCACGTAAGG
Nr1d1	forward	CTGGAGGGCTGCAGTATAGC
	reverse	GTCCAGGGTCGTCATGTCTT
Dbp	forward	AATGACCTTTGAACCTGATCCCGCT
	reverse	GCTCCAGTACTTCTCATCCTTCTGT

© 2020 by the authors. Submitted for possible open access publication under the terms and conditions of the Creative Commons Attribution (CC BY) license (http://creativecommons.org/licenses/by/4.0/).
